# Withdrawing versus Withholding Treatments in Medical Reimbursement Decisions: A Study on Public Attitudes

**DOI:** 10.1177/0272989X241258195

**Published:** 2024-06-24

**Authors:** Liam Strand, Lars Sandman, Emil Persson, David Andersson, Ann-Charlotte Nedlund, Gustav Tinghög

**Affiliations:** Swedish National Centre for Priorities in Health, Department of Health, Medicine, and Caring Sciences, Linköping University, Sweden; Swedish National Centre for Priorities in Health, Department of Health, Medicine, and Caring Sciences, Linköping University, Sweden; Department of Management and Engineering, Linköping University, Sweden; Department of Management and Engineering, Linköping University, Sweden; Swedish National Centre for Priorities in Health, Department of Health, Medicine, and Caring Sciences, Linköping University, Sweden; Swedish National Centre for Priorities in Health, Department of Health, Medicine, and Caring Sciences, Linköping University, Sweden; Department of Management and Engineering, Linköping University, Sweden

**Keywords:** equivalence thesis, experiment, health policy, priority setting

## Abstract

**Background:**

The use of policies in medical treatment reimbursement decisions, in which only future patients are affected, prompts a moral dilemma: is there an ethical difference between withdrawing and withholding treatment?

**Design:**

Through a preregistered behavioral experiment involving 1,067 participants, we tested variations in public attitudes concerning withdrawing and withholding treatments at both the bedside and policy levels.

**Results:**

In line with our first hypothesis, participants were more supportive of rationing decisions presented as withholding treatments compared with withdrawing treatments. Contrary to our second prestated hypothesis, participants were more supportive of decisions to withdraw treatment made at the bedside level compared with similar decisions made at the policy level.

**Implications:**

Our findings provide behavioral insights that help explain the common use of policies affecting only future patients in medical reimbursement decisions, despite normative concerns of such policies. In addition, our results may have implications for communication strategies when making decisions regarding treatment reimbursement.

**Highlights:**

- We explore public’ attitudes toward withdrawing and withholding treatments and how the decision level (bedside or policy level) matters.
- People were more supportive of withholding medical treatment than of withdrawing equivalent treatment.
- People were more supportive of treatment withdrawal made at the bedside than at the policy level.
- Our findings help clarify why common-use policies, which impact only future patients in medical reimbursement decision, are implemented despite the normative concerns associted with thesepolicies.

## Graphical abstract

**Graphical abstract:**
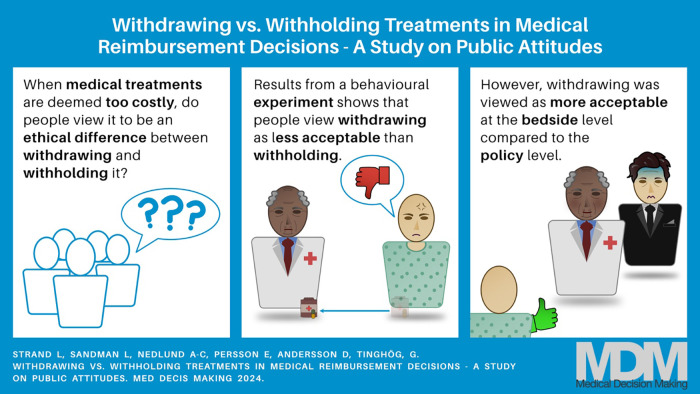

When a patient initially gains access to a medicine, later deemed cost-ineffective, resulting in a decision to deny it to subsequent patients within the health care system, a delicate ethical dilemma arises: should the original patient maintain continued access to the medication? In rationing dilemmas like this, reimbursement agencies may adopt policies where existing situations follow an old rule while a new rule is applied to all future cases.^1^ Such policies suggest that treatment is not withdrawn from patients with initial access to the medication but is withheld from future patients who suffer from the same condition. This introduces an ethical distinction between withdrawing and withholding treatments. However, it remains unclear whether the use of such policies reflects a substantive difference in ethical views between cases or signifies cases of bounded ethicality.^2^ In this study, we conduct an experiment to explore whether people express a view of an ethical difference between withdrawing and withholding treatments in this type of reimbursement context. Moreover, we explore if these attitudes vary across the bedside and policy levels.

Polices that imply ethical inequivalence between withdrawing and withholding medical treatments have sparked intense debate (see, e.g., Wester et al.,^1^ Sandman and Liliemark^3^). Critics argue that these policies result in 1) differential treatment for patients with similar medical needs; 2) a first-come, first-served approach to treatment; and 3) inefficient use of health care resources when non–cost-effective treatments are not withdrawn. As a result, arguments have been made in favor of treating withdrawing and withholding as ethically equivalent actions in health care rationing. Despite these arguments, policies in which patients already on treatment are treated differently continue to be used in practice (see, e.g., National Institute for Health and Care Excellence^4^), indicating a perceived ethical distinction when reimbursing medical treatments.

There is a notable reluctance among many to consider withdrawing and withholding treatments as equivalent in practice.^5,6^ When considering the reimbursement of medical treatments (hereafter referred to as the “reimbursement context”), studies indicate public support for the use of policies in which only future patients are affected^7^ and for continuing reimbursement of treatments already in use, rather than reimbursing new treatments.^8^ However, these studies have not explicitly examined public attitudes towards the ethical difference between withdrawing and withholding treatments; thus, these indications remain uncertain. In contrast, studies conducted on rationing for end-of-life treatments (hereafter referred to as the “end-of-life context”) consistently show that practitioners prefer withholding life-saving treatments over withdrawing them.^6^ In one survey, the discrepancy in acceptance was as large as 82 percentage points.^9^ However, it is unclear whether the results from the end-of-life context can be generalized to the reimbursement context. Nonetheless, the previous research forms the basis for our first hypotheses (H1):

H1: The acceptance toward limiting patients’ access to treatments will be lower when withdrawing treatments compared with withholding treatment.

An interview study conducted within the reimbursement context showed that physicians and patient organization representatives perceive the bedside and policy levels as ethically significant factors when considering the withdrawal and withholding of treatments. Participants emphasized the importance of the patient-physician relationship and communication at the bedside level, while at the policy level, they highlighted the need for guidelines on how to withdraw and withhold treatments as well as a transparent process for the reimbursement decision.^5^ Experimental studies have shown that the public is more inclined to ration medical resources at the policy level rather than at the bedside level,^10,11^ indicating a stronger orientation toward efficiency at the policy level. However, those previous studies have only studied the context of withholding treatments at the bedside and policy level. Thus, it is unclear whether their results extend to a withdrawing context. Nonetheless, these findings form the basis for our second hypothesis (H2):

H2: The acceptance toward limiting patients’ access to treatments will be lower at the bedside level compared with the policy level.

In this study, we present the results from an experiment in which we explore whether people express an ethical difference between withdrawing and withholding treatments in a reimbursement context. We also explore if these attitudes vary between bedside-level and policy-level decisions and whether the decision level affects preferences for withdrawing and withholding treatments differently. Two preregistered hypotheses are tested: H1, the acceptance toward limiting patients’ access to treatments will be lower when withdrawing treatments compared with withholding treatment, and H2, the acceptance toward limiting patients’ access to treatments will be lower at the bedside level compared with the policy level.

## Methods

### Participants and Procedure

The study was preregistered. The preregistration together with data and analysis codes can be accessed via this repository: https://osf.io/sqd3e/. The online experiment was programmed in Qualtrics, and the data were collected in March 2023. A total of 1,404 English-speaking participants (59% female, mean age 39.90 [*s* = 13.13] y) were recruited from Prolific.^12^ Participants were randomized into 1 of 4 conditions that were similar in terms of age and sex, indicating successful randomization (see Supplementary Table S1). Eighty-seven percent passed the attention check included, and following our preregistered analyses plan, we conducted robustness checks by excluding participants who failed the attention check. See the Supplementary Materials for experimental materials including attention check.

### Study Design

The study was originally designed as a 2 × 2 between-subjects experiment in which participants were randomized into 1 of 4 conditions: 1) withdrawing treatment at the policy level, 2) withholding treatment at the policy level, 3) withdrawing treatment at the bedside level, and 4) withholding treatment at the bedside level. We had an unfortunate programming error in condition 4), which meant that we had to remove it from the analysis.^i^ The exclusion of condition 4) withholding treatment at the bedside level means that we were not able to test the third research question outlined in our preregistration, which related to the interaction effect between rationing type and decision level. This research question was, however, explorative in nature, given the absence of direction hypothesis in our initial planning. Importantly, we can still test our 2 main research questions and hypotheses by using condition 1) withdrawing and policy level as a control condition to test how rationing type and decision level affect preferences for limiting patients’ access to treatments.

Table 1 shows the rationing dilemma presented to the participants across experimental conditions. In the control condition, the treatment was being withdrawn and concerned the assessment of a dilemma presented at the policy level with a public health agency. After reading the dilemma, participants in the control condition were asked whether a policy to withdraw patients’ medicine would be acceptable/unacceptable.

**Table 1:** Rationing Dilemma Across Experimental Conditions

Control Condition	Withholding Condition	Bedside Condition
A public health agency is deciding which medical treatments patients will and will not get access to. Currently, there are patients suffering from a serious disease and in need of a specific medicine. Without access to this medicine, the patients’ quality of life will be negatively affected.	A public health agency is deciding which medical treatments patients will and will not get access to. Currently, there are patients suffering from a serious disease and in need of a specific medicine. Without access to this medicine, the patients’ quality of life will be negatively affected.	**A physician** is deciding which medicines patients will and will not get access to. Currently, there are patients suffering from a serious disease and in need of a specific medicine. Without access to this medicine, the patients’ quality of life will be negatively affected.
However, an independent assessment of the costs and benefits associated with the medicine has concluded that it is not deemed cost-effective, meaning that the resources spent on the medicine could be better used elsewhere in the health care system. The recommendation following the assessment of cost-effectiveness is therefore to deny patients access to the medicine.	However, an independent assessment of the costs and benefits associated with the medicine has concluded that it is not deemed cost-effective, meaning that the resources spent on the medicine could be better used elsewhere in the health care system. The recommendation following the assessment of cost-effectiveness is therefore to deny patients access to the medicine.	However, an independent assessment of the costs and benefits associated with the medicine has concluded that it is not deemed cost-effective, meaning that the resources spent on the medicine could be better used elsewhere in the health care system. The recommendation following the assessment of cost-effectiveness is therefore to deny patients access to the medicine.
The public health agency is faced with a decision regarding the medicine. Should it be rationed, meaning that the medicine would be withdrawn from patients who are currently undergoing and experiencing the benefits of the medical treatment. If the medicine is not withdrawn from patients, cutbacks instead need to be done on treatments elsewhere in the health care system. In your opinion, would a policy to withdraw the medicine from patients be acceptable/unacceptable?	The public health agency is faced with a decision regarding the medicine. Should it be rationed, meaning that the medicine would be **withheld** from patients who are currently **seeking and would experience** the benefits of the medical treatment. If the medicine is not **withheld** from patients, cutbacks instead need to be done on treatments elsewhere in the health care system. In your opinion, would a policy to **withhold** the medicine from patients be acceptable/unacceptable?	**The physician** is faced with a decision regarding the medicine. Should it be rationed, meaning that the medicine would be withdrawn from patients who are currently undergoing and experiencing the benefits of the medical treatment. If the medicine is not withdrawn from patients, cutbacks instead need to be done on treatments elsewhere in the health care system. In your opinion, would **a decision from the physician to** withdraw the medicine from patients be acceptable/unacceptable?

In the control condition, treatment is withdrawn at a policy level. The difference in the withholding and bedside conditions compared with the control condition is **bolded**. The participants were randomized to 1 of these conditions.

The withholding condition was identical to the control condition, except that the patients were “seeking and would experience the benefits of the medical treatment” instead of “undergoing and experiencing the benefits of the medical treatment.” In addition, the term *withdraw* was throughout the vignette substituted with *withhold*.

The bedside condition was identical to the control condition, except that instead of assessing a dilemma at the policy level for a public health agency, the dilemma was presented at the bedside level for a physician. Respondents were then asked whether a physician deciding to withdraw the patients’ medicine would be acceptable/unacceptable instead of a policy to withdraw the medicine.

In addition to a binary outcome variable of whether it is acceptable/unacceptable to limit the patients’ access to a treatment, we also measured acceptability on a Likert scale, ranging from 1 = *completely unacceptable* to 7 = *completely acceptable*, to test for more nuanced changes in attitudes. See the Supplementary Materials for a full transcript of the survey.

### Analysis

We used a linear probability model to test whether being in the withholding or bedside condition affected the probability of viewing it as acceptable to limit the patients’ access to treatment due to lack of cost-effectiveness in the moral dilemma. First, to test our hypotheses, we used a binary outcome variable (acceptable/unacceptable) as the dependent variable with the 2 treatments as independent variables. To test the robustness of the results, we added our control variables (age, gender, education level) to the regression model. Thereafter, we further tested the robustness by running the same regression, with control variables, but excluding participants who had failed the attention check. Lastly, we repeated the analysis but used the 7-point Likert-type scale outcome variable instead of a binary outcome variable. The analysis was conducted in *R*.

### Deviation from Preregistration

The programming error that led us to exclude condition 4 from the analysis forced us to make some inevitable deviations from the preset analysis plan. In the preregistration, we stated that we would test the interaction effect between rationing type and decision level. Due to the failed condition, this test of interaction between conditions was not possible to carry out. For the same reason, we had to change our main analysis from a 2-way analysis of variance to a linear regression analysis (ordinary least squares). Other than that, we followed our preset analysis plan.

## Results

**Figure 1:**
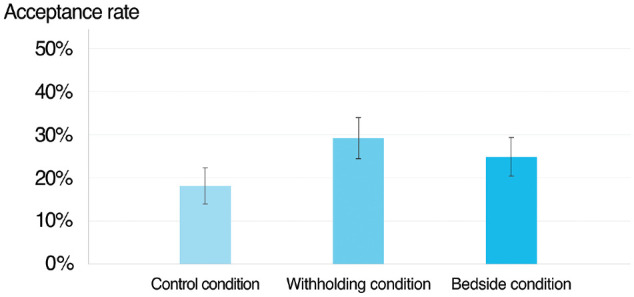
Share of participants viewing rationing as acceptable (versus unacceptable) across experimental conditions The error bars display 95% confidence intervals.

Figure 1 shows the acceptance for limiting treatment separated by condition. There was an 11-percentage-point higher support for withholding treatments (29.19% acceptance) compared with when withdrawing the same treatment (18.26% acceptance). The difference was statistically significant (*t* = 3.46, *P* < 0.001) and in line with hypothesis 1, predicting a higher support for withholding treatment compared with withdrawing the same treatment in the control condition. Figure 1 also shows that there was a 7-percentage-point higher support for withdrawing treatments at the bedside level (25.00% acceptance) compared with the policy level (18.26% acceptance). The difference was also statistically significant (*t* = 2.16, *P* = 0.031) but in the opposite direction to our prestated hypothesis 2, predicting that support for rationing would be lower at the bedside compared with the policy level.

We tested the robustness of the result by conducting linear regressions (Table 2). The regression shows that our results for withholding (*t* = 3.62, *P* < 0.001) and bedside (*t* = 2.28, *P* = 0.023) were robust when controlling for demographics (see model 1). The regression analysis in Table 2 also shows that being male was associated with being 12 percentage points more likely to view it as acceptable to limit the patients’ access to treatments compared with females (*t* = 4.31, *P* < 0.001). Participants who had completed at most a secondary education were 12 percentage points less likely to view it as acceptable to limit the patients’ access to treatments (*t* = −3.69, *P* < 0.001) compared with participants with a higher educational level. There was no statistically significant difference between further education level and higher. Age had no statistically significant effect.

**Table 2:** Regression Analysis on Acceptance for Limiting Patients’ Access to Treatments

	Model 1			Model 2		
	Beta	SE	*P* Value	Beta	SE	*P* Value
Withholding condition	0.11	0.03	<0.001	0.11	0.03	<0.001
Bedside condition	0.07	0.03	0.023	0.06	0.03	0.058
Age	0.00	0.00	0.26	0.00	0.00	0.41
Male	0.12	0.03	<0.001	0.11	0.03	<0.001
<Secondary education	−0.12	0.03	<0.001	−0.11	0.03	0.001
Further education	−0.04	0.03	0.188	−0.03	0.03	0.30
Intercept	0.12	0.05	0.015	0.13	0.05	0.012
	*n* = 1,067			*n* = 943		

All regressions are ordinary least squares with robust standard errors. Acceptance for limiting patients’ access to treatments was measured as either being acceptable (=1) or unacceptable (=0). Higher education (>3 y) is the reference group for education. Model 2 is identical to model 1 but excludes participants who failed an attention check.

When excluding participants who failed the attention check (model 2), the effect of withholding remained robust (*t* = 3.34, *P* < 0.001). However, the bedside versus policy effect was no longer statistically significant when excluding subjects who failed the attention check (*t* = 1.90, *P* = 0.058), but the effect remained similar in size and direction. Interestingly, there was no statistically significant difference between conditions when we switched the binary yes/no outcome variable to the 7-point Likert-scale outcome variable (see Supplementary Table S3). Nonetheless, the direction of the effects was consistent with the main analysis.

## Discussion

In light of the ethical and practical challenges associated with reimbursement decisions, leading to the use of policies in which only future patients are denied treatment, we conducted a preregistered experiment on the matter. Our objective was to test how attitudes toward limiting patients’ access to treatments due to lack of cost-effectiveness are affected by rationing type (withdrawing/withholding treatments) and decision level (bedside level and policy level). The results showed that participants were more supportive of limiting patients’ access to treatment when presented as withholding treatments compared with withdrawing treatments, showing an 11-percentage-point difference. This result has implications for policy makers, suggesting some public support for policies that differentiate between withdrawing and withholding treatments in the reimbursement context. Results also show that participants were 7 percentage points more supportive of limiting patients’ access to treatments by withdrawing treatment at the bedside level compared with the policy level. This finding may have implications for communication strategies when making decisions regarding treatment reimbursement. Despite physicians and patient organization representatives commonly expressing the view that the allocation of scarce resources is primarily a policy-level decision,^5^ our results suggest that this does not necessarily translate to a preference for policy makers to make these decisions.

Our first main finding indicating more support for withholding treatment over withdrawing the same treatment aligns with our initial prediction (H1). This study contributes to previous research on similar matters in 2 important ways. First, we directly test variations in attitudes toward withdrawing and withholding treatment within a reimbursement context, moving beyond reliance on implicit measures. Second, the results empirically substantiate the qualitatively generated hypotheses of previous studies,^5^ subjecting them to testing through a controlled experiment. Our results reinforce beliefs that individuals are more inclined to limit access to care by opting to withhold treatment rather than withdrawing the same treatment from patients currently undergoing care.

The overall acceptance of rationing for cost-effectiveness in the study was notably low, with 29% or less endorsing such measures. This is striking, especially considering the context of a UK sample, in which the public health care system frequently restricts access to treatments based on cost-effectiveness (as in National Institute for Health and Care Excellence^4^). The timing of our study coincided with a period marked by decreased support for the UK health care system,^13^ potentially contributing to the general lack of endorsement for rationing decisions. In addition, our deliberate emphasis on cost-effectiveness considerations in the study design likely contributed to the overall lack of public support for rationing decision (see, e.g., Tinghög and Västfjäll^14^). This stands in contrast to prior research on the rationing of end-of-life care,^6^ where support has been higher and cost-effectiveness was not emphasized to the same extent as a primary rationale for rationing.

### Differences between the Bedside and Policy Level

Participants had higher acceptance for withdrawing treatments at the bedside level compared with withdrawing treatments at the policy level. This finding is in sharp contrast to the results reported in previous experimental studies comparing rationing at the bedside and policy level.^10,11^ However, previous studies have predominantly centered on rationing decisions that specifically involve the withholding of treatment (and not the withdrawing of treatments). Thus, their results may not be generalizable to withdrawing of treatment.

Although the higher acceptance of withdrawing treatment at the bedside level was not in line with our prestated hypothesis, in hindsight, this finding appears reasonable. Following the work on the importance of construal level,^15^ it is possible that the bedside level is seen as more concrete due to the closer social distance between the patient and the physician, whereas the policy level is seen as more concrete due to the greater social distance between the patient and the policy maker. Previous studies^16,17^ have demonstrated that people often prefer efficiency over equality when either the policy or principle for rationing is more concretely formulated. Therefore, if the bedside level is perceived as more concrete, individuals may prioritize efficiency, potentially leading to a greater inclination to accept rationing based on cost-effectiveness at the bedside level, as observed in the current study.

Another potentially contributing factor for why we see higher acceptance of withdrawing treatments at the bedside level compared with the policy level is related to the legitimacy of the decision maker. Previous studies have shown that the public is more supportive of physicians being responsible for rationing situations compared with other professions such as ethicists, health economists, or politicians.^17^ Therefore, the preference for the bedside level over the policy level may be attributed to the perceived legitimacy of the decision maker rather than solely being influenced by psychological mechanisms such as social distance.

A third potentially contributing factor for why we get a “reversed bedside effect” could be that participants mistakenly thought that the physician (as opposed to policy makers) withdraw the treatment due to beneficence of the patient and not due to cost-effectiveness reasons and therefore viewed it as more acceptable at the bedside compared with the policy level. Regardless of the underlying mechanism for why participants were more supportive of bedside-level decisions to withdraw treatment than for analogous decisions made at the policy level, our study provides empirical evidence that rationing preferences is likely to be affected by the decision level, even when the outcome for the patients and the motivation behind rationing decisions are identical.

### Limitations

As with any study, there are some limitations worth noting. First, a programming error forced us to deviate from our prestated analysis plan, and we were not able to test the interaction effect of rationing type and decision level. Importantly, we were still able to test our 2 main preregistered research questions and hypotheses. To ensure credibility and transparency of the research, we explicitly report all deviations from our analysis plan. Moreover, all data, including that from the failed condition, is available on the project’s repository https://osf.io/sqd3e/. Second, our use of an online sample of UK residents may affect the generalizability of the results in an international context. However, previous comparisons have shown that online samples often show good data quality in comparison with other recruitment methods. We used Prolific, which has been shown to provide high-quality data in recent investigations.^18^ In addition, our experimental design allowed us to control the environment and ensure a high internal validity.

### Future Research

To better understand the difference between bedside and policy levels, future research should test whether the legitimacy of the decision maker influences decisions to ration treatments at the bedside and policy level. It would also be valuable to investigate other underlying mechanisms affecting preferences for withdrawing and withholding treatments, for example, examining the sunk cost fallacy, in which withdrawing a treatment can be perceived as discontinuing an investment,^19^ or studying the opportunity cost neglect, in which people may not consider the opportunity cost in priority setting.^20,21^ In our study, we explicitly presented the opportunity cost, but removing that information would allow for testing opportunity cost neglect. Another potential mechanism at play that we did not test in this study is the status quo or omission bias,^22,23^ in which maintaining the status quo (i.e., keeping access to treatment for withdraw and not gaining access to it for withhold) would be preferable to taking action to change the status quo. Furthermore, it would be valuable to investigate other contextual factors, such as those identified by previous qualitative research.^5^ For instance, examining the impact of factors such as prior agreements with patients regarding future treatment access, availability of alternative treatments, or the process through which patients obtain access to treatments, would provide a more comprehensive understanding of preferences for the ethical dilemma of withdrawing and withholding treatments within a reimbursement context.

## Conclusions

We conducted a preregistered experiment examining public attitudes toward limiting patients’ access to treatments in a reimbursement context. Our results provide empirical evidence that people consider withholding treatment as more acceptable than withdrawing it. Therefore, rationing policies suggesting an ethical distinction between withdrawing and withholding treatments may also be publicly accepted. This result could then explain why policies in which only future patients are affected are used when deciding not to reimburse medical treatments despite normative concerns associated with such policies. In addition, our findings indicate that the withdrawal of treatments at the bedside level is perceived as more acceptable than at the policy level. This suggests that people prioritize efficiency at the bedside level and may attribute greater legitimacy to physicians in making rationing decisions compared with policy makers.

## Supplemental Material

sj-csv-2-mdm-10.1177_0272989X241258195 – Supplemental material for Withdrawing versus Withholding Treatments in Medical Reimbursement Decisions: A Study on Public Attitudes Supplemental material, sj-csv-2-mdm-10.1177_0272989X241258195 for Withdrawing versus Withholding Treatments in Medical Reimbursement Decisions: A Study on Public Attitudes by Liam Strand, Lars Sandman, Emil Persson, David Andersson, Ann-Charlotte Nedlund and Gustav Tinghög in Medical Decision Making

sj-docx-3-mdm-10.1177_0272989X241258195 – Supplemental material for Withdrawing versus Withholding Treatments in Medical Reimbursement Decisions: A Study on Public Attitudes Supplemental material, sj-docx-3-mdm-10.1177_0272989X241258195 for Withdrawing versus Withholding Treatments in Medical Reimbursement Decisions: A Study on Public Attitudes by Liam Strand, Lars Sandman, Emil Persson, David Andersson, Ann-Charlotte Nedlund and Gustav Tinghög in Medical Decision Making

sj-rmd-1-mdm-10.1177_0272989X241258195 – Supplemental material for Withdrawing versus Withholding Treatments in Medical Reimbursement Decisions: A Study on Public Attitudes Supplemental material, sj-rmd-1-mdm-10.1177_0272989X241258195 for Withdrawing versus Withholding Treatments in Medical Reimbursement Decisions: A Study on Public Attitudes by Liam Strand, Lars Sandman, Emil Persson, David Andersson, Ann-Charlotte Nedlund and Gustav Tinghög in Medical Decision Making

## Notes

i. The failed condition used the word *withdraw* in 1 of 3 places where it should have been *withhold*, thus making it impossible to interpret the result in that condition. For full transparency, the data from the failed conditions are also available at https://osf.io/sqd3e/.
