# Supplementary material for: Withdrawing versus Withholding Treatments in Medical Reimbursement Decisions: A Study on Public Attitudes
Source: Med Decis Making. 2024 Jun 24;44(6):641–8. doi: 10.1177/0272989X241258195 (PMC11346081; doi:10.1177/0272989X241258195)
Supplement: sj-docx-3-mdm-10.1177_0272989X241258195 – Supplemental material for Withdrawing versus Withholding Treatments in Medical Reimbursement Decisions: A Study on Public Attitudes [file sj-docx-3-mdm-10.1177_0272989X241258195.docx]

Supplementary Materials – Exploring Attitudes to the Use of Grandfather Clauses in Health Care Rationing: A Pre-registered Experiment

# Supplementary Statistical Analysis

As seen in Table S1**,** 24.27% believed that it was acceptable to limit the patient’s access to treatments in the rationing dilemma. Therefore, there was generally a low acceptance towards limiting patients’ access to treatments. Furthermore, the average age was 39.90 (SD=13.13), 41.38% were male, the education level was high as 55.84% had completed higher education and 25.07% had completed further education. Moreover, 87.25% of the participants completed the attention check, and the average response time was 11.46 minutes.

A One-Way ANOVA show that mean age was not statistically different between the four experiment conditions (D(3, 1400), *F*=0.05, *p*=0.98). Furthermore, Chi-square tests show that there were no statistically significant differences for either gender (χ^2^(3, 1404)=1.87, *p*=0.60) or education level ((χ^2^(6, 1404)=6.81, *p*=0.34). A One-Way ANOVA also show that the mean response time did not statistically differ between the four experiment conditions (D(3, 1400), *F*=0.96, *p*=0.41). When using the full dataset, there was a statistically significant difference in completion of the attention check ((χ^2^(3, 1404)=8.86, *p*=0.031). However, this was expected as the programming error invalidates the attention check, as the attention check was dependent on withdrawing/withholding being clear.

| Table S1: Descriptive Statistics for the Complete Dataset | | | | | |
| --- | --- | --- | --- | --- | --- |
| Condition | Control | Bedside | Withhold | Failed |  |
| Rationing Type | Withdraw | Withdraw | Withhold | (Withhold) | All |
| Abstractness | Policy | Bedside | Policy | Bedside | All |
| Acceptability to Limit Access, % (n) | 18.26% (63) | 25% (88) | 29.19% (108) | 36.50%^*^ (123) | 27.20% (382) |
| Age, mean (SD) | 39.96 (13.56) | 40.08 (12.93) | 39.85 (12.43) | 39.69 (13.69) | 39.90 (13.13) |
| Male, % (n) | 44.35% (153) | 40.91% (144) | 39.46% (146) | 40.95% (138) | 41.38% (581) |
| Primary Education, % (n) | 0.87 % (3) | 0.85% (3) | 0.27% (1) | 0.59% (2) | 0.64% (9) |
| Secondary Education, % (n) | 16.81% (58) | 17.61% (62) | 17.84% (66) | 21.66% (73) | 18.45% (259) |
| Further Education, % (n) | 29.28% (101) | 24.43% (86) | 24.32% (90) | 22.26% (75) | 25.07% (352) |
| Higher Education, % (n) | 53.04% (183) | 57.10% (201) | 57.57% (213) | 55.49% (187) | 55.84% (784) |
| Completed Attention Check, % (n) | 87.25% (301) | 86.65% (305) | 91.08% (337) | 83.68% (282) | 87.25% (1225) |
| Response time in minutes, mean (SD) | 11.54 (6.97) | 11.42 (7.01) | 11.84 (7.23) | 10.99 (5.39) | 11.46 (6.71) |
| n | 345 | 352 | 370 | 337 | 1404 |
| Note: * This result cannot be interpreted due to the programming error. |  |  |  |  |  |
|  | | | | | |

Table S2 displays the descriptive statistics for the conditions used in the study after excluding participants from the failed condition. We tested if there remained any statistically significant in completion of the attention check, which there were no longer any differences (χ^2^( 2, 1067)=4.09, *p*=0.130). Moreover, as none of the other statistical analysis for differences in demographics was statistically significant for all four conditions, there was no need to test if there were any differences for the remaining three conditions.

| **Table S2: Descriptive Statistics in the Experiment** | | | | |
| --- | --- | --- | --- | --- |
| Condition | Control | Withhold | Bedside |  |
| Rationing Type | Withdraw | Withhold | Withdraw | All |
| Abstractness | Policy | Policy | Bedside | All |
| Acceptability to Limit Access, % (n) | 18.26% (63) | 29.19% (108) | 25% (88) | 24.27% (259) |
| Age, mean (SD) | 39.96 (13.56) | 39.85 (12.43) | 40.08 (12.93) | 39.96 (12.96) |
| Male, % (n) | 44.35% (153) | 39.46% (146) | 40.91% (144) | 41.52% (443) |
| At most Secondary Education, % (n) | 17.68% (61) | 18.11% (67) | 18.47% (65) | 18.09% (193) |
| Further Education, % (n) | 29.28% (101) | 24.32% (90) | 24.43% (86) | 25.96% (277) |
| Higher Education, % (n) | 53.04% (183) | 57.57% (213) | 57.10% (201) | 55.95% (597) |
| Completed Attention Check, % (n) | 87.25% (301) | 91.08% (337) | 86.65% (305) | 88.38% (943) |
| Response time in minutes, mean (SD) | 11.54 (6.97) | 11.84 (7.23) | 11.42 (7.01) | 11.61 (7.07) |
| n | 345 | 370 | 352 | 1067 |

Table S3 displays the same main regression analysis as in the article (Table 2) but with a 7-point Likert scale outcome variable. Compared to the main analysis, neither the effect of withholding (*t*=1.43, *p*=0.154) nor bedside (*t*=1.67, *p*=0.094) were statistically significant when controlling for demographics. The effects for withholding (*t*=1.12, *p*=0.26) and bedside (*t*=1.13, *p*=0.26) remained statistically insignificant when excluding participants who failed the attention check (Model S2).

| **Table S3: Regression Analysis on Acceptance for Limiting Patients' Access to Treatments with Likert Outcome Variable** | | | | | | |
| --- | --- | --- | --- | --- | --- | --- |
|  | Model S1 - Demographics | | | Model S2 - Attention | | |
|  | Beta | SE | p-value | Beta | SE | p-value |
| Withholding | 0.15 | 0.11 | 0.154 | 0.13 | 0.12 | 0.26 |
| Bedside | 0.19 | 0.11 | 0.094 | 0.14 | 0.12 | 0.26 |
| Age | 0.01 | 0.00 | 0.048 | 0.01 | 0.00 | 0.41 |
| Male | 0.24 | 0.09 | 0.008 | 0.19 | 0.10 | 0.057 |
| <Secondary Education | -0.41 | 0.12 | <0.001 | -0.40 | 0.13 | 0.002 |
| Further Education | -0.25 | 0.11 | 0.018 | -0.22 | 0.11 | 0.050 |
| Intercept | 2.78 | 0.17 | <0.001 | 2.80 | 0.18 | <0.001 |
|  | n=1067 | | | n=943 | | |
| Note: All regressions are ordinary least square with robust standard errors. Acceptance for limiting patients’ access to treatments was measured on a 7-point Likert scale with 7=completely acceptable and 0=completely unacceptable. Higher education (>3 years) is the reference group for education. Model S2 is identical to Model S1 but excludes participants who failed an attention check. | | | | | | |

# Power Calculations

We conducted power calculations based on the original study design. As specified by Lakens and Caldwell (1), it is better to base the power calculation for 2x2 interaction in ANOVA by the expected pattern of means than the Cohen’s f. In our case, we expected an ordinal interaction, which increases the needed sample size (1). Therefore, we assumed the following means and standard deviation:

- Withdraw*High Abstractness = 0.10
- Withhold*High Abstractness = 0.90
- Withdraw*Low Abstractness = 0.09
- Withhold*Low Abstractness = 0.70
- Standard deviation = 0.5 since it is a binary outcome variable

These means led to a large effect size for withdraw/withholding (Cohen’s f = 0.71) which is plausible given the results of Wilkinson and Savulescu (2). Thereafter, the means led to a small effect size for abstractness (Cohen’s f = 0.1), which also is plausible given previous research (3, 4). Most importantly, it led to an ordinal- and small sized interaction effect (Cohen’s f = 0.10). Note: >0.02 small effect size, >0.15 medium effect size, >0.35 large effect size Cohen (5).

Inserting these values in the ANOVA_exact()-function from Lakens and Caldwell (1) Superpower R-package (run install. packages("Superpower") in R), we found that a sample size of at least 292 per condition (n=1168) is needed for a 2x2 between-subjects ANOVA with 90% power with 5% alpha.

# The Original Study Design

## The 4 Conditions

| Withdrawing | Withdrawing | Withholding | Withholding |
| --- | --- | --- | --- |
| High Abstract Policy Level | Low Abstract Bedside Level | High Abstract Policy Level | Low Abstract Beside Level |
| **A public health agency** is deciding which medical treatments patients will and will not get access to. Currently, there are patients suffering from a serious disease and in need of a specific medicine. Without access to this medicine, the patients’ quality of life will be negatively affected. | **A physician** is deciding which medicines patients will and will not get access to. Currently, there are patients suffering from a serious disease and in need of a specific medicine. Without access to this medicine, the patients’ quality of life will be negatively affected. | **A public health agency** is deciding which medical treatments patients will and will not get access to. Currently, there are patients suffering from a serious disease and in need of a specific medicine. Without access to this medicine, the patients’ quality of life will be negatively affected. | **A physician** is deciding which medicines patients will and will not get access to. Currently, there are patients suffering from a serious disease and in need of a specific medicine. Without access to this medicine, the patients’ quality of life will be negatively affected |
| However, an independent assessment of the costs and benefits associated with the medicine has concluded that it is not deemed cost-effective, meaning that the resources spent on the medicine could be better used elsewhere in the health care system.  The recommendation following the assessment of cost-effectiveness is therefore to deny patients access to the medicine. | However, an independent assessment of the costs and benefits associated with the medicine has concluded that it is not deemed cost-effective, meaning that the resources spent on the medicine could be better used elsewhere in the health care system.  The recommendation following the assessment of cost-effectiveness is therefore to deny patients access to the medicine. | However, an independent assessment of the costs and benefits associated with the medicine has concluded that it is not deemed cost-effective, meaning that the resources spent on the medicine could be better used elsewhere in the health care system.  The recommendation following the assessment of cost-effectiveness is therefore to deny patients access to the medicine. | However, an independent assessment of the costs and benefits associated with the medicine has concluded that it is not deemed cost-effective, meaning that the resources spent on the medicine could be better used elsewhere in the health care system.  The recommendation following the assessment of cost-effectiveness is therefore to deny patients access to the medicine. |
| The **public health agency** is faced with a decision regarding the medicine. Should it be rationed, meaning that the medicine would be *withdrawn* from patients currently *undergoing* and *experiencing* the benefits of the medical treatment. If the medicine is not *withdrawn* from patients**,** cutbacks instead need to be done on treatments elsewhere in the health care system. | **The physician** is faced with a decision regarding the medicine. Should it be rationed, meaning that the medicine would be *withdrawn* from patients who are currently *undergoing* and *experiencing* the benefits of the medical treatment. If the medicine is not *withdrawn* from patients**,** cutbacks instead need to be done on treatments elsewhere in the health care system. | The **public health agency** is faced with a decision regarding the medicine. Should it be rationed, meaning that the medicine would be *withheld* from patients who are currently *seeking* and *would experience the* benefits of the medical treatment. If the medicine is not *withheld* from patients**,** cutbacks instead need to be done on treatments elsewhere in the health care system. | **The physician** is faced with a decision regarding the medicine. Should it be rationed, meaning that the medicine would be *withheld* from patients who are currently *seeking* and *would experience the* benefits of the medical treatment. If the medicine is not *withheld* from patients, cutbacks instead need to be done on treatments elsewhere in the health care system. |
| In your opinion, would **a policy to** *withdraw* the medicine from patients be acceptable/unacceptable? | In your opinion, would **a decision from the physician to** *withdraw* the medicine from patients be acceptable/unacceptable? | In your opinion, would **a policy to** *withhold* the medicine from patients be acceptable/unacceptable? | In your opinion, would **a decision from the physician to** *withhold^[[1]](#footnote-1)^* the medicine from patients be acceptable/unacceptable? |

# Experimental instructions (part 1 – vignette)

*INSTRUCTIONS FOR ALL CONDITIONS*

Welcome!

If you agree to participate in this study, we will ask you several questions related to your attitudes towards prioritisation of limited health care resources in different circumstances.

Your answers will be treated so no unauthorised persons access them. All publication of this research will only report results on an aggregated level or completely anonymized examples which will not identify you.

Participation in the study should take about 10 minutes and you will receive a participation fee of £2 for completing the entire study.

Please note: During the survey, there will be an attention check to ensure that you are paying attention to the study instructions.

There are no foreseeable risks to your participation in this study. By clicking the button below, you indicate that you understand the information that was presented and that your participation is voluntary, and you may withdraw your consent and discontinue participation in the project at any time.

- I consent to participate in this study

Please enter your Prolific ID

________________________________________________________________

[NEW SCREEN]

## [Control condition]

On the next screen you will be presented with a hypothetical but plausible description of a health care dilemma.

You will be asked to imagine a public health agency and you will assess the acceptability of a described policy.

Keep in mind that there is no right or wrong answer to the described scenario. The questions might be perceived as difficult but try to express your honest opinion on the described situation in your answer.

Please consider the details and information within the scenario carefully prior to giving your answers to each question. You will be asked a small number of basic comprehension questions throughout the survey to assess information retention.

[NEW SCREEN]

A public health agency is deciding which medical treatments patients will and will not get access to. Currently, there are patients suffering from a serious disease and in need of a specific medicine. Without access to this medicine, the patients’ quality of life will be negatively affected.

However, an independent assessment of the costs and benefits associated with the medicine has concluded that it is not deemed cost-effective, meaning that the resources spent on the medicine could be better used elsewhere in the health care system.

The recommendation following the assessment of cost-effectiveness is therefore to deny patients access to the medicine.

The public health agency is faced with a decision regarding the medicine. Should it be rationed, meaning that the medicine would be withdrawn from patients who are currently undergoing and experiencing the benefits of the medical treatment. If the medicine is not withdrawn from patients, cutbacks instead need to be done on treatments elsewhere in the health care system.

In your opinion, would a policy to withdraw the medicine from patients be acceptable/unacceptable?

- Unacceptable
- Acceptable

To help us better understand the strength of your opinion, how acceptable/unacceptable would a policy to withdraw the patients’ medicine be?

- 1=Completely unacceptable
- 2
- 3
- 4
- 5
- 6
- 7=Completely acceptable

## [Withholding condition]

On the next screen you will be presented with a hypothetical but plausible description of a health care dilemma.

You will be asked to imagine a public health agency and you will assess the acceptability of a described policy.

Keep in mind that there is no right or wrong answer to the described scenario. The questions might be perceived as difficult but try to express your honest opinion on the described situation in your answer.

Please consider the details and information within the scenario carefully prior to giving your answers to each question. You will be asked a small number of basic comprehension questions throughout the survey to assess information retention.

[NEW SCREEN]

A public health agency is deciding which medical treatments patients will and will not get access to. Currently, there are patients suffering from a serious disease and in need of a specific medicine. Without access to this medicine, the patients’ quality of life will be negatively affected.

However, an independent assessment of the costs and benefits associated with the medicine has concluded that it is not deemed cost-effective, meaning that the resources spent on the medicine could be better used elsewhere in the health care system.

The recommendation following the assessment of cost-effectiveness is therefore to deny patients access to the medicine.

The public health agency is faced with a decision regarding the medicine. Should it be rationed, meaning that the medicine would be withheld from patients who are currently seeking and would experience the benefits of the medical treatment. If the medicine is not withheld from patients, cutbacks instead need to be done on treatments elsewhere in the health care system.

In your opinion, would a policy to withhold the medicine from patients be acceptable/unacceptable?

- Unacceptable
- Acceptable

To help us better understand the nuances of your opinion, how acceptable/unacceptable would a policy to withhold the patients’ medicine be?

- 1=Completely unacceptable
- 2
- 3
- 4
- 5
- 6
- 7=Completely acceptable

## [Bedside condition]

On the next screen you will be presented with a hypothetical but plausible description of a health care dilemma.

You will be asked to imagine a physician and you will assess the acceptability of a described behaviour.

Keep in mind that there is no right or wrong answer to the described scenario. The questions might be perceived as difficult but try to express your honest opinion on the described situation in your answer.

Please consider the details and information within the scenario carefully prior to giving your answers to each question. You will be asked a small number of basic comprehension questions throughout the survey to assess information retention.

[NEW SCREEN]

A physician is deciding which medicines patients will and will not get access to. Currently, there are patients suffering from a serious disease and in need of a specific medicine. Without access to this medicine, the patients’ quality of life will be negatively affected.

However, an independent assessment of the costs and benefits associated with the medicine has concluded that it is not deemed cost-effective, meaning that the resources be^[[2]](#footnote-2)^ spent on the medicine could be better used elsewhere in the health care system.

The recommendation following the assessment of cost-effectiveness is therefore to deny patients access to the medicine.

The physician is faced with a decision regarding the medicine. Should it be rationed, meaning that the medicine would be withdrawn from patients who are currently undergoing and experiencing the benefits of the medical treatment. If the medicine is not withdrawn from patients, cutbacks instead need to be done on treatments elsewhere in the health care system.

In your opinion, would a decision from the physician to withdraw the medicine from patients be acceptable/unacceptable?

- Unacceptable
- Acceptable

To help us better understand the nuances of your opinion, how acceptable/unacceptable would a decision from the physician to withdraw patients’ medicine be?

- 1=Completely unacceptable
- 2
- 3
- 4
- 5
- 6
- 7=Completely acceptable

## [Failed condition]

On the next screen you will be presented with a hypothetical but plausible description of a health care dilemma.

You will be asked to imagine a physician and you will assess the acceptability of a described behavior.

Keep in mind that there is no right or wrong answer to the described scenario. The questions might be perceived as difficult but try to express your honest opinion on the described situation in your answer.

Please consider the details and information within the scenario carefully prior to giving your answers to each question. You will be asked a small number of basic comprehension questions throughout the survey to assess information retention.

[NEW SCREEN]

A physician is deciding which medicines patients will and will not get access to. Currently, there are patients suffering from a serious disease and in need of a specific medicine. Without access to this medicine, the patients’ quality of life will be negatively affected.

However, an independent assessment of the costs and benefits associated with the medicine has concluded that it is not deemed cost-effective, meaning that the resources spent on the medicine could be better used elsewhere in the health care system.

The recommendation following the assessment of cost-effectiveness is therefore to deny patients access to the medicine.

The physician is faced with a decision regarding the medicine. Should it be rationed, meaning that the medicine would be withheld from patients who are currently seeking and would experience the benefits of the medical treatment. If the medicine is not withheld from patients, cutbacks instead need to be done on treatments elsewhere in the health care system.

In your opinion, would a decision from the physician to withdraw^[[3]](#footnote-3)^ the medicine from patients be acceptable/unacceptable?

- Unacceptable
- Acceptable

To help us better understand the strength of your opinion, how acceptable/unacceptable would a decision from the physician to withhold patients’ medicine be?

- 1=Completely unacceptable
- 2
- 3
- 4
- 5
- 6
- 7=Completely acceptable

# Attention check

*INSTRUCTIONS FOR ALL CONDITIONS*

[NEW SCREEN]

In the previous question on how the health care provides treatments, what would happen with the patients’ treatment?

- It would be withdrawn (correct for control and bedside condition)
- It would be withheld (correct for withhold and failed condition)
- It would be financed
- It would be reimbursed

# Experimental instructions (part 2 – demographics)

*INSTRUCTIONS FOR ALL CONDITIONS*

[NEW SCREEN]

How old are you?

(The participants dragged a slider ranging from 18-100 with intervals of to indicate their age)

What’s your gender?

- Male
- Female

What’s your highest completed education?

- Primary education
- Secondary education (completed year 13)
- Further education (post-secondary and tertiary education)
- Higher education (at least Bachelor’s degree)

[NEW SCREEN]

We thank you for your time spent taking this survey.

Your response has been recorded.

# References

1. Lakens D, Caldwell AR. Simulation-Based Power Analysis for Factorial Analysis of Variance Designs. Advances in Methods and Practices in Psychological Science. 2021;4(1) doi:10.1177/2515245920951503

2. Wilkinson D, Savulescu J. A Costly Separation between Withdrawing and Withholding Treatment in Intensive Care. Bioethics. 2014;28(3):127-37. doi:10.1111/j.1467-8519.2012.01981.x

3. Tinghög G, Strand L. Public Attitudes Toward Priority Setting Principles in Health Care During COVID-19. Frontiers in Health Services. 2022;2. doi:10.3389/frhs.2022.88650

4. Li M, DeWitt J. Equality by principle, efficiency by practice: How policy description affects allocation preference. Interdisciplinary perspectives on fairness, equity, and justice. Cham, Switzerland: Springer International Publishing; 2017. p. 67-91.

5. Cohen J. Statistical Power Analysis for the Behavioral Sciences. 2013. doi:10.4324/9780203771587

1. This is where the programming error happened and instead stood withdraw. [↑](#footnote-ref-1)
2. This was a writing error, as the word should simply not have been there. [↑](#footnote-ref-2)
3. This where the programming error happened, as it should have stood withhold here. [↑](#footnote-ref-3)
